# Editorial: Cellular Mechanisms During Normal and Abnormal Craniofacial Development

**DOI:** 10.3389/fcell.2022.872038

**Published:** 2022-03-08

**Authors:** Poongodi Geetha-Loganathan, John Abramyan, Marcela Buchtová

**Affiliations:** ^1^ Department of Biological Sciences, SUNY Oswego, Oswego, NY, United States; ^2^ Department of Natural Sciences, University of Michigan–Dearborn, Dearborn, MI, United States; ^3^ Department of Experimental Biology, Faculty of Science, Masaryk University, Brno, Czechia; ^4^ Laboratory of Molecular Morphogenesis, Institute of Animal Physiology and Genetics, Czech Academy of Sciences, Brno, Czechia

**Keywords:** palatogenesis, tooth development, eye, neural crest, craniofacial

## Introduction

**FIGURE 1:**
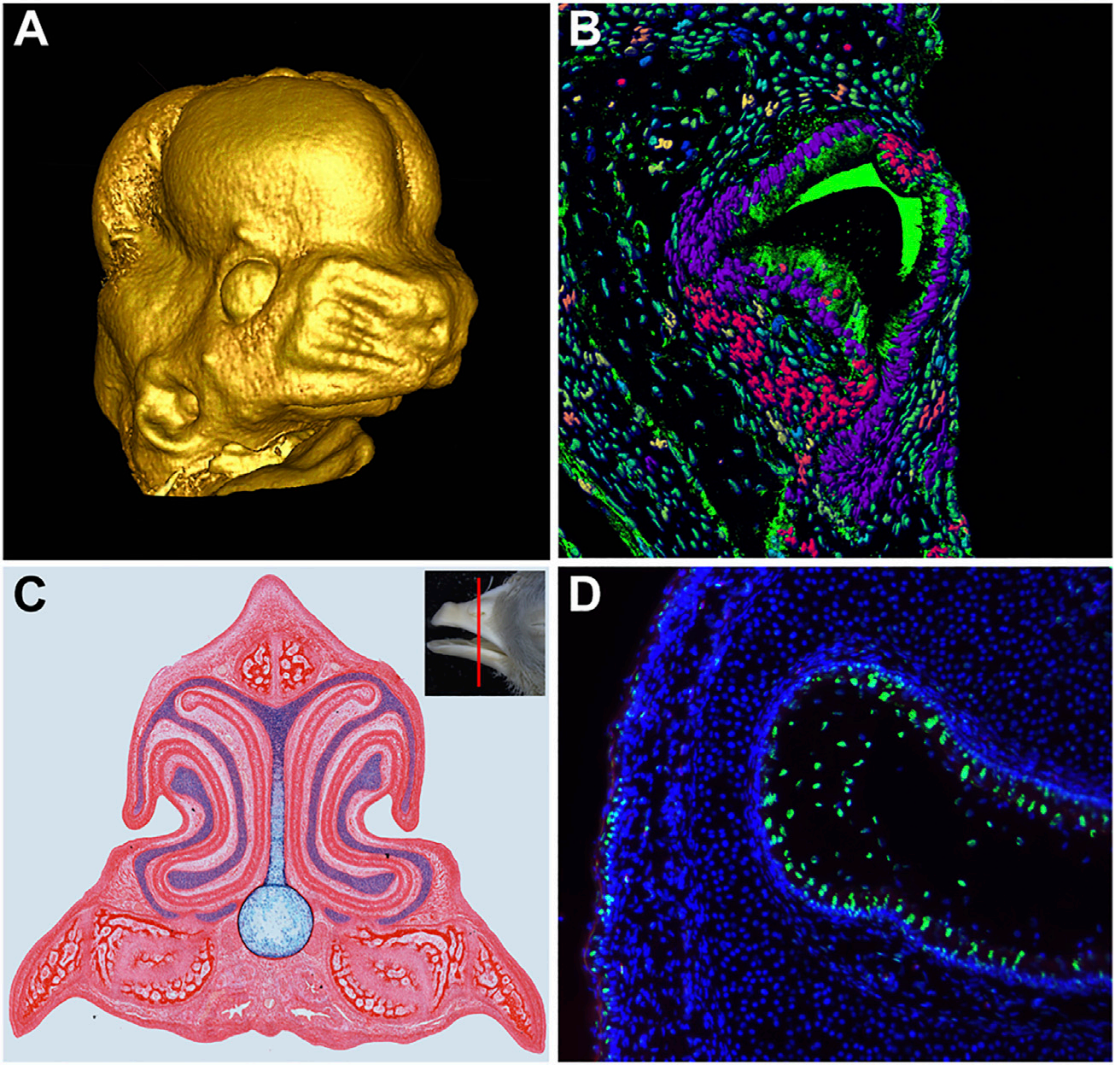
Development of Craniofacial Structures. **(A)** MicroCT scan of embryonic day 13 mouse embryo face, reconstructed using MicroView software. **(B)** Transverse section of the chameleon tooth. Artificial colors of nuclei were produced in software IMARIS followed by segmentation and analysis for cell nuclei shape differences. **(C)** Transverse section of Hamburger and Hamilton (1951) stage 40 chicken embryo beak stained with picrosirius red to highlight skeletal and soft tissue and alcian blue to highlight cartilage. **(D)** Apoptotic signal (green) in the developing nasal cavity of a Hamburger and Hamilton (1951) stage 42 chicken embryo.

Embryonic craniofacial development involves a series of cellular processes that drive patterning, outgrowth, and fusion of a number of independently forming components. The progression of growth and morphogenesis relies on cellular mechanisms such as differentiation, proliferation, migrations, transformation, and apoptosis to form the correct shape and structure in the developing embryo (Figure 1). During craniofacial development, the aforementioned processes are spatiotemporally constrained, allowing for multiple mechanisms within a relatively small region in order to create complex and intricate structures. Failure at any stage risks considerable consequences for the embryo, ranging from slight defects in craniofacial patterning to total inviability.

The current Research Topic “*Cellular Mechanisms During Normal and Abnormal Craniofacial Development*” intends to examine and collate the most up-to-date studies on the cellular processes that drive embryonic craniofacial morphogenesis. In recent years, scientists have taken great strides towards attaining a better understanding of the cellular mechanisms involved in the fundamental aspects of craniofacial development. Seemingly disparate fields such as computer sciences, mathematics, tomographic and microscopic imaging, biochemistry, and molecular biology have come together to greatly enhance our understanding of how individual cells influence the overall patterning and morphogenesis of the embryonic face.

This Research Topic includes 21 papers that focus on the cellular processes involved in the development of a variety of craniofacial structures such as palate, teeth, eyes, craniofacial muscles, as well as their contribution to associated developmental defects. Additionally, the importance of interaction between neural tissue and forming face is introduced and discussed.

## Cellular Processes in Craniofacial Structures Shaping and Patterning

Murrilo-Rincón and Kaucka reviewed the complexity of craniofacial structure development from a cellular perspective. They focused on the cell fate specification in craniofacial structures from the emergence of neural crest cells up to the establishment of facial geometry. Individual or collective cell migration, cellular interactions, and physical forces generated by morphogenetic events, all contribute to the establishment of the face as well as intra- and inter-species morphological variability. The importance of molecular regulation, including epigenetic events and alteration of signaling center activity, is introduced, in addition to the different types of craniofacial defects they can lead to, ranging from subtle changes of facial symmetry to severe lethal conditions.

In vertebrates, morphogenesis of the upper jaw and face is regulated by cellular interactions between the brain, facial ectoderm, and neural crest mesenchyme; mediated by several morphogenetic signals including wingless (WNT) (Geetha-Loganathan et al., 2009; Rickbourg et al., 2020), sonic hedgehog (SHH) (Hu et al., 2015), fibroblast growth factor (FGF) (Hu et al., 2003), and bone morphogenetic protein (BMP) (Barlow and Francis-West, 1997). During embryonic development, the relationship between brain and face has also received some attention, with disruption of signals altering patterning of both structures (Richbourg et al., 2020).

Since Wnt signaling from the facial ectoderm and forebrain is required to regulate the outgrowth of facial prominences, activating and inhibiting the expression of canonical Wnt signaling in these regions causes malformation of facial structures, including cleft lip (Green et al., 2019). In this topic, Marchini et al. illustrate the interactions between the brain and face by activating *WNT3a* and inhibiting Wnt signaling with *DKK1* in the neural crest of developing chicken embryos. Their study uncovered that *WNT3a* activation expands the shape of the frontonasal ectodermal zone (FEZ) along with the brain and face during early face formation, whereas changes in the brain and face are decoupled during later developmental stages, supporting the palimpsest model for morphological integration (Hallgrímsson et al., 2009).

Mutations in Ras-related C3 botulinum toxin substrate 1 (Rac1) encoding a small GTPase have been shown to be involved in brain tumorigenesis and abnormal development (Khalil and El-Sibai, 2012). Gahankari et al. have elucidated the activity of *Rac1* signaling during the development of the midbrain dopaminergic neurons (mDA). They have created Rac1CA;Wnt1-Cre2 mutant mice with specific activation of Rac1^G12v^ mutant in neural crest cell derivatives. These mutants are characterized by an enlarged midbrain due to increased proliferation and abnormal migration of neural cells, disrupting localization of mDA in the ventral side.

## Palatogenesis

In the mammalian embryo, the assembly of the maxillary region is a precarious period of development. Improper fusion of the upper lip and/or secondary palate is exceedingly common, with the incidence of cleft lip/palate (CLP) in humans occurring at a rate of one in 750 newborns, among the most frequent birth defects worldwide (Kousa and Schutte, 2016). Palate development consists of the growth, elevation, and fusion of the palatal shelves, starting at 6–8 weeks of gestation in humans (Mossey et al., 2009). Cleft palate (CP) is etiologically complex and heterogeneous, occurring in isolated and syndromic cases, and involving a combination of genetic and environmental factors, with many cases remaining unexplained (Mossey et al., 2009; Buser and Pohl, 2015).

Manuscripts in the recent topic focused on a wide range of developmental processes ranging from undersized palatal shelves to the retention of the medial epithelial seam (MES). One of the disorders associated with cleft palate is KGB syndrome. The suspected cause of KGB syndrome is the *Ankrd11* gene, a chromatin regulator associated with neural stem cell fates. Here, Roth et al. report that mice with heterozygous deletion of *Ankrd11* in neural crest cells display craniofacial defects that mirror KBG syndrome, including hypoplastic palatal shelves, which the study found was due to a regional decrease in mesenchymal proliferation. Ultimately, their study identifies *Ankrd11* as a critical regulator of intramembranous ossification and palate development and suggests that *Ankrd11* neural crest knockout mice may serve as novel models for KBG syndrome.

Keeping the focus on cell proliferation in palatal shelves, Yoshioka et al. studied the effect of all-trans retinoic acid (atRA) on microRNA (miR) expression during palatogenesis. atRA is used in the treatment of skin diseases and cancers, and yet is a teratogen known to cause cleft palate. The authors found that excessive atRA induces expression of MicroRNA-4680-3p, which suppresses genes crucial for palate development, leading to decreased cell proliferation in human embryonic palatal mesenchymal cells. This study is important in not only highlighting the involvement of miRs in palatogenesis but also elucidating a molecular relationship between miRs and palatal clefting.

In case shelves reach an appropriate size, they have to undergo elevation from a vertical position to horizontal; yet another potential pitfall. On occasion, palatal shelves fail to horizontalize in a timely manner. Bukova et al. observed this phenotype in their study of the CRISPR/Cas9 WIZ knockout mouse model. The authors found that loss of *Wiz* causes disruption of histone methylation known to be driven by the G9a/GLP methylation complex. Altered histone methylation causes delayed palatal shelf horizontalization, which resulted in CP since the medial edge epithelium of the palatal shelves misses the window of competence to fuse, even if horizontalization eventually occurs.

Even after the horizontalization and approach of palatal shelves, the fusion process is complex and often disrupted. Van der Woude syndrome (VWS) is associated with palatal clefting; with almost three-quarters of cases caused by mutations in the transcription factor IRF6. IRF6 is a master regulator balancing keratinocyte proliferation and differentiation, with *Irf6*-deficient mice displaying disorganized oral epithelium. In their study, Degen et al. identified a novel heterozygous VWS-causing *IRF6* mutation that lacks part of its protein-binding domain and entire C-terminus. This truncated *IRF6* copy undergoes nonsense-mediated mRNA decay, leading to haploinsufficiency. The remaining *IRF6* copy can still control gene regulatory networks but does not meet the threshold required for proper periderm arrangement, resulting in pathological epithelial fusions between tissues in the oral cavity, thereby interfering with shelf lift, outgrowth, and fusion.

In studying the mechanism of IRF6-caused CP, Girousi et al. found that lack of IRF6 disrupts epithelial homeostasis by altering colony morphology, migration pattern, and differentiation potential of cultured postnatal skin- and oral mucosa-derived keratinocytes. Colonies appeared scattered with less stable cell-cell contacts and a significant number of single and enlarged cells. Wound-healing experiments showed IRF6-deficient keratinocytes preferentially moving as single cells, with random and undirected migration, potentially explaining the appearance of disrupted epithelium in IRF6 mutants. The proteomic analysis further supported these results by finding that most of the differentially expressed proteins in the absence of IRF6 were associated with differentiation, cell-cell adhesion, and immune response.

Assuming epithelia do manage to fuse, the disintegration of the MES needs to occur in order for fusion to be complete. Verheijen et al. studied the interplay between CXCL12 ligand and CXCR4 receptor in association with the breakdown of the MES and osteogenesis during palatal fusion in mice. They conclude that CXCL12 and *Sox9* expression by the disintegrating MES and osteogenic centers in the fusing palatal shelves could promote the maturation of immature CXCR4-positive osteoblasts. Furthermore, *Sox9* progenitors appear to be important in maintaining the CXCR4-positive osteoblast pool to drive osteogenesis as well as potentially regulate MES disintegration through EMT.

One of the studies focused on the soft palate, which lies posterior to the hard palate and consists of a muscular structure that also undergoes fusion (Danescu et al., 2015). Deng et al. utilized *in vivo* overexpression of *Noggin*, an antagonist of the BMP pathway, and disrupted development of muscles, tendons, and aponeuroses in the soft palate. Suppression of BMP signaling in palatal mesenchyme inhibited the differentiation of aponeurosis and tendons, which resulted in the hypoplasia of palatal muscles, along with decreased mesenchymal cell proliferation and survival due to disruption of epithelial *Shh* expression. Interestingly, the authors found that impaired myogenesis and tenogenesis were not involved in the clefting of the soft palate. Instead, clefting was attributed to the reduced cell proliferation and survival caused by the interrupted Shh-Gli1 signaling, and the impaired development of the aponeurosis.

## Odontogenesis

Papers on tooth development focused on different aspects of cell interactions during odontogenesis. The role of cell death during tooth morphogenesis and its key effect on surrounding cell populations are reviewed by Abramyan et al. The distribution of apoptotic cells during odontogenesis is compared in different vertebrate lineages from fish to mammals. Apoptosis is introduced in light of active cellular events affecting molecular signaling and behavior in neighboring cells. Apoptosis-associated molecular signaling, including factors from intrinsic and extrinsic apoptotic pathways, as well as microRNAs and autophagic signaling are described during dental cell differentiation and morphogenesis. Apoptotic pathway disruption, alterations in apoptotic cell distribution, and dysregulation of apoptosis in mutant mouse models with mutations in non-apoptotic genes, were found to contribute to the disruption of odontogenesis.

To identify the transcriptomic characteristics of dental cells in mouse incisors, Chiba et al. performed single-cell RNA-sequencing. They focused on the identification of epithelial dental subpopulations with the aim of uncovering novel marker genes for each dental cell type that eventually contributes to enamel production. They identified two subclusters of secretory ameloblasts with a distinctive expression of *Dspp* and *Ambn*, with pseudo-time analysis finding that *Dspp*-positive cells differentiate into *Ambn*-positive ameloblasts. Their study can be used as a large resource of transcriptome data for further analyses of molecular regulation controlling the formation of enamel.

The tight junctions play a key role in ameloblast differentiation and enamel production in mouse incisors. Wang et al. examined expression patterns of claudin family members during odontogenesis and found *Cldn1* and *Cldn10* to be highly expressed in the dental epithelium. *Cldn10* was found as a novel stratum intermedium marker and a role for *Cldn10* in the regulation of dental epithelial cell differentiation during amelogenesis was confirmed by functional analysis.

The ecto-endodermal boundary in the oral cavity of axolotl was analyzed by Soukup et al. in order to determine the potential source of instructive factors that induce odontogenesis. The ecto-endodermal boundary runs through the tooth fields so that individual tooth germs display differential origins from ectodermal, endodermal, or ecto-endodermal epithelial cells. Common tooth-competent zones were found in the roof of the oral cavity and one in the mouth floor, from which five pairs of tooth fields are arranged into typical tetrapod dental patterns. By using GFP-labeled oral ectoderm, they followed the spatial relationships of early initiated teeth for individual dental fields and uncovered that the first tooth usually develops in close association to the ecto-endodermal boundary.

## Eye Development

The literature on the contribution of neural crest cells to the anterior segment of the eye was reviewed by Williams and Bohnsack. They cover molecular regulation of eye development and associated congenital eye diseases, with a special focus on neural crest cell-related signaling. Rare ocular diseases such as Axenfeld-Rieger syndrome, Peters anomaly, and primary congenital glaucoma are discussed in light of key genes for neural crest cell specification or migration. Theories of disease mechanisms are also debated as an understanding of neural crest cell signaling disruption may improve the management of degenerative ocular diseases.

Disruption of eye development can also occur during the closure of the optic fissure, resulting in ocular coloboma. Cellular processes and molecular signaling, including basement membrane dynamics, cell behavior, and the fate of cells contributing to the fusion are discussed by Chan et al. Recently, a large number of model organisms spanning fish, avian, and mammalian species have been used to uncover potential avenues for new research strategies and testing of factors, which can help to prevent the occurrence of fusion defects.

## Development of Branchiomeric Muscles

Head and trunk skeletal muscles have distinct functions and embryonic origins. Trunk muscles are responsible for locomotion while craniofacial muscles control eye movement, facial expression, and mastication. Cranial neural crest cells migrate to the branchial arches (BA) and along with mesoderm cells regulate the development of BA muscles (Lescroart et al., 2010).

Retinoic acid (RA) signaling is shown to be involved in the development of craniofacial muscles (Wang et al., 2019) but the mechanisms used by RA in regulating BA muscles are not well understood. Wang et al. found that RA exposure induces malformation of branchiomeric muscles in mice, this is associated with increased cell death of branchial mesodermal cells. Also, the development of hypoplastic craniofacial muscles in RA treated embryos is due to reduced expression of *Pitx2, Tbx1*, and *MyoD,* in the first and second BA. Further, over-expression of RA signaling inhibited *Dlx5* and *Dlx6* expression in cranial neural crest cells, which altered their communications with branchial mesoderm cells.

The neck muscles in the region connecting the head and trunk originate from the posterior BA and are associated with processes such as respiration, vocalization, swallowing, and mobility of the head (Heude et al., 2018). In the trunk, the myogenic precursor cells migrate from the ventrolateral lip of the dermomyotome to the myogenic regions within limbs, diaphragm, and tongue (Masyuk and Brand-Saberi, 2015). CXCR4/SDF-1 axis is shown to regulate the migration of muscle progenitor cells and play a crucial role in the trunk, neck, and head muscle development. Yahya et al. reviewed the roles of the CXCR4/SDF-1 axis in the development of facial and neck muscles. The key discussions include the precursors of facial expression and mastication muscles that originate from mesoderm cells of BA1 and BA2, whereas the progenitors for non-branchiomeric head muscles come from the prechordal mesoderm, and progenitor mesoderm cells of the head and somites contribute to non-somatic neck muscles. CXCR4/SDF-1 axis is involved in the migration of progenitors for the formation of branchiomeric muscles and may be responsible for muscular dystrophies.

## Clinical Aspects of Disruptions of Cellular Processes

Craniofacial anomalies (CFAs) observed in 0.2% of all newborns can appear like CLP or as a syndromes involving a mutation in a single gene or chromosomal aberrations. For some CFAs, the genetic background has yet to be identified. Although CFAs can be treated with surgery, it is regularly associated with complications with feeding, speech, hearing, dentition, and healing (Hortis-Dzierbicka et al., 2012). Studies on facial development in animals and humans reveal discrepancies and often do not allow all findings in animals to be transferred to humans. To overcome this issue Parisi et al. established a living cell repository of the Cranio-/orofacial region as a clinically relevant cell model to learn CFAs, with patient-derived cells obtained from discarded tissue biopsies.

Primary cilia dysfunction or ciliopathies are often associated with craniofacial pleiotropic phenotypes. Craniofacial ciliopathic phenotypes include CLP, craniosynostosis, and micrognathia (Schock and Brugmann, 2017). Understanding the significance of the basal body is necessary to gain knowledge for therapeutic treatments because most of the genes associated with ciliopathies encode proteins specific to basal bodies, centrosomes, and centriolar satellites. Ciliogenesis is regulated by a centriolar protein encoded by the C2 domain containing 3 centriole elongation regulators (C2cd3) and mutation of this gene causes Oral-Facial-Digital syndrome (OFD) in humans. Chang et al. characterized three novel C2cd3 mouse models, a conditional knockout, and two novel CRISPR-targeted lines targeting regions in the divergent C2CD3N-C2 domain or PKC-C2 domains. Also, by examining conditional alleles with two neural crest-specific drivers, the authors showed that C2CD3N-C2 is more important for craniofacial development compared to C-terminal PKC-C2 domains.

The pinna or auricle of the ear is affected in many craniofacial syndromes including Lacrimo-auriculo-dento-digital syndrome (LADD) caused by defects in FGF signaling (Teshima et al., 2016). Zhang et al. used an FGF10 knockout mouse to elucidate the mechanisms behind the pinna defects in LADD syndrome. Loss of function of Fgf10 in mice results in smaller, cup-shaped pinnae, similar to dimorphisms seen in LADD syndrome. The phenotype is driven by increased proliferation on the outer part of the pinna together with decreased proliferation in the inner part, causing inward bending.
